# Anaesthesia for cleft lip surgeries in a resource poor setting: techniques, outcome and safety

**DOI:** 10.11604/pamj.2018.31.105.15513

**Published:** 2018-10-12

**Authors:** Olumide Adeleke Akitoye, Babatunde Oludare Fakuade, Thomas Oseghae Owobu, Akinwale Adeyemi Efunkoya, Adetokunbo Rafel Adebola, Sunday Olusegun Ajike

**Affiliations:** 1 Department of Anaesthesia, College of Health Sciences,University of Abuja, FCT, Abuja, Nigeria; 2 Department of Dental and Maxillofacial Surgery, Federal Medical Centre, Gombe State, Nigeria; 3 Department of Dental and Maxillofacial Surgery, Federal Medical Centre,Nguru, Yobe State, Nigeria; 4 Department of Dental and Maxillofacial Surgery, Aminu Kano Teaching Hospital, Kano State, Nigeria; 5 Department of Dental and Maxillofacial Surgery, Ahmadu Bello University Teaching Hospital, Zaria, Kaduna State, Nigeria

**Keywords:** Anaesthesia, cleft lip, techniques, setting

## Abstract

**Introduction:**

Cleft lip and palate is one of the more common congenital malformation and the most common craniofacial anomalies in children. The treatment is expensive and requires specialised care. Access to this care in middle and low income countries is compounded by socioeconomic status of patients and their relation and also the inadequacy of expertise in medical personnel and infrastructure. Objective: the study aimed to review the techniques of anaesthesia used in a low resource setting in terms of the techniques, outcome, and safety.

**Methods:**

This is a retrospective review of 79 cases done in a resource poor setting. Information regarding the patients, surgeries and modes of anaesthesia were retrieved from the case notes.

**Results:**

A total of 62 patients were operated with incomplete cleft accounting for 37 (59.7%), complete 23(37.1%), and 2 (3.2%) as bilateral. Forty-six (74.2%) of patients had their surgery done with ketamine anaesthesia without endotracheal intubation, 14 (22.6%) had regional anaesthesia and 2 patients (3.2%) had general anaesthesia with endotracheal intubation.

**Conclusion:**

This study demonstrates that with careful planning and expertise, cleft lip repair can be done safely in resource poor setting.

## Introduction

Cleft lip and palate has a huge impact on the life of an individual and their family. It is one of the more common congenital malformation and the most common craniofacial anomalies in children [1]. A child is born with a cleft somewhere in the world every 2 minutes according to a WHO study published in 2001 [2]. The prevalence of cleft lip, with or without an associated cleft palate, is 0.1% in the general population [3]. The treatment of orofacial cleft anomaly is expensive and requires years of specialized care [3, 4]. Though successful treatment of the cosmetic and functional aspects of orofacial cleft anomalies is now possible, presentation for surgery is late in low and middle income countries due to low socioeconomic status of patients and their relation [4]. This is further compounded by the inadequacy of expertise in the surgical procedure, competent anaesthetists, and non-availability of necessary equipment [5]. The civil war that ravaged Sierra Leone from 1991 to 2002 destroyed the country's infrastructure including its health systems. Anaesthesia for cleft lip and palate surgery is challenging to the anaesthetist [6]. Two safe modes of anaesthesia described are general anaesthesia with endotracheal intubation and regional anaesthesia [3, 7]. General anaesthesia is mostly used for patients in the paediatric age group and endotracheal intubation is necessary to secure and protect the airway because the surgeon shares the same field with the anaesthetist. Regional anaesthesia is mostly used in patients in whom cooperation could be sought. It could be by infraorbital nerve block, dorsal nasal block or peri-incisional infiltration [3, 8]. Bearing in mind the challenges involved in the anaesthesia for cleft lip surgery which include difficult airway, inadvertent extubation, kinking of endotracheal tube, aspiration of blood and secretions, laryngospasm, etc [7, 9, 10], this study intend to review a number of cases done in a resource poor setting enumerating the mode of anaesthesia, safety, complications, and recovery profile of the patients.

## Methods

This is a retrospective study of anaesthesia for the surgical repair of cleft lip in the country of Sierra Leone by a team of Nigerian surgeons and anaesthetist on a smile train international mission. The information is as recorded in the patients' case notes. A total of seventy-nine (79) patients presented during the mission. The patients were evaluated clinically for fitness for surgery and any coexisting congenital malformations. Information regarding patient's weight, written consent, preoperative fasting guideline, mode of anaesthesia, excessive bleeding, adverse events during surgical procedure, intra and postoperative complications like fever, excessive secretion, hypoxia, vomiting, bleeding, and need for intubation were retrieved. The need for additional anaesthetic or analgesic, conversion to general anaesthesia from regional technique was also retrieved. No routine pre-operative laboratory test was done. Standard preoperative fasting guidelines were observed based on records. Three modes of anaesthesia were recorded: regional anaesthesia for patients above the age of 9 years (Group A), a modified general anaesthesia with supplemental peri-incisional infiltration for patients aged 9years and below with unilateral cleft lip (Group B), and general anaesthesia with endotracheal intubation (Group C) for patient with bilateral cleft lip. Dental preparation of lidocaine hydrochloride with adrenaline (36mg + 18µg) in 1.8ml cartridges were used in all the modes of anaesthesia. In both groups A and B, patients were left to breathe in room air. All patients had intravenous access with infusion of 4.3% Dextrose in 0.18 Saline or 0.9% Saline as applicable. Monitoring was with the aid of precordial stethoscope, and lifebox pulse oximeter.

**Group A:** Patients had peri-incisional infiltration of the dental preparation of lidocaine with adrenaline. Surgeries were done with the patients in the sitting position, awake and breathing room air.

**Group B:** Patients had intramuscular ketamine at a dose of 10-12.5 mg/kg mixed with atropine at a dose of 0.01-0.03mg/kg. Supplemental peri-incisional infiltration of the dental preparation of lidocaine with adrenaline was instituted after induction. Saline wet gauze was placed in the buccal pouch. Surgery was in the supine position with a shoulder roll and head ring in place.

**Group C:** Patients had general anaesthesia with endotracheal intubation. Induction was inhalational with halothane in oxygen at 6L/min to achieve hypnosis. Intubation was facilitated with suxamethonium 1.5mg/kg given intravenously. Maintenance of anaesthesia was with halothane at 1% concentration in oxygen at 6L/min. Patient also had peri-incisional infiltration of the dental preparation of lidocaine with adrenaline. The surgery was also performed in the supine position with shoulder roll and head ring in place. Patients were allowed to breathe spontaneously. Fever was defined as temperature of more than 38.5°C, hypoxia as percentage saturation of oxygen of less than 90%, and excessive bleeding and secretion as those that required the use of suction machine. Duration of surgery was defined as the time from knife on skin to the end of last stitch.

## Results

**Figure 1:**
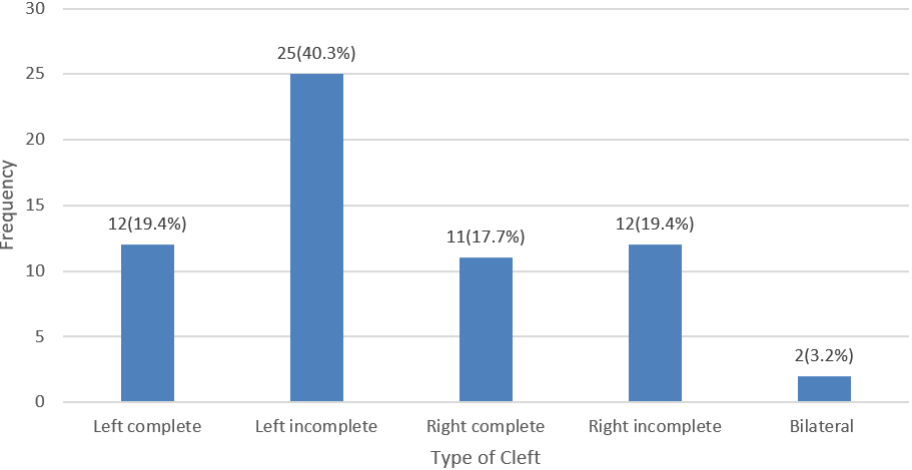
Type of cleft

A total of 79 patients presented for surgery during the mission which lasted 10 days. Sixty-two patients comprising 34 males (54.8%) and 28 females (45.2%) were operated and hence studied. The remaining 17 patients had isolated palatal cleft which could not be repaired because of limited facility to guaranty safe surgery and anaesthesia. The age distribution of the patients is presented in Table 1. Of the 62 operated patients, 37(59.7%) had left unilateral cleft lip with 25(40.3%) left incomplete and 12(19.4%) left complete cleft lip. 11(17.7%) had right complete cleft lip and 12 (19.4%) with right incomplete cleft lip while only 2(3.2%) had bilateral cleft lip (Figure 1). Forty six (46) patients (74.2%) were anaesthetized with ketamine at a dose of 10-12.5mg/kg combined with atropine 0.01-0.03mg/kg given intramuscularly without endotracheal intubation, while 14 (22.6%) had regional anaesthesia and only 2 patients (3.2%) had general anaesthesia with endotracheal intubation. The mean duration of surgery was 34.2minutes (median 34.0minutes). The only complication recorded is fever which occurred in 6 patients (9.7%) who had ketamine anaesthesia (Table 2).

**Table 1:** Age of patient

	Frequency (n=62)	Percent (%)
**Age Group (mean±SD)**	5.7±8.5	
<1year	19	30.6
1-5years	27	43.5
6-10years	5	8.1
11-15years	4	6.5
16-20years	3	4.8
>=20	4	6.5

Range 3months-43years SD= Standard Deviation

**Table 2:** Mode of anaesthesia and complication

Mode of anaesthesia	Complications		Chi-square	P-value
	Fever n=6 n(%)	Nil n=56 n(%)		
GA with Intubation	0	2(3.2)	2.311	0.315
Ketamine	6(9.7)	40(64.5)		
Regional	0	14(22.6)		

## Discussion

Modern anaesthetic techniques in cleft surgeries involve the use of expensive anaesthetic agents, bulky machines, and continuous flow of anaesthetic gases [11]. This is largely because of the shared field situation in which the surgeon and the anaesthetist work in the same region of the body i.e. the oral cavity. All the patients operated had their surgery for the first time (primary repair). Majority of the patients (34(54.8%)) were males while there were 28(45.2%) females. This finding is in agreement with the work done by Jindal *et al* and Kulkarni *et al* [12, 13]. Studies have also shown the predominance of left unilateral cleft lip [3, 5, 6]. This correlates well with the finding of 37(59.7%) unilateral cleft lip in this study. Ketamine with atropine, given intramuscularly, was the main technique of Anaesthesia recorded. This technique was employed because of the non-availability of sophisticated gadgets and also the limitation of the supply of oxygen. In this study, this technique was used for all patients aged 3 months to 9 years with unilateral cleft lip (both complete and incomplete). This is in contrast to the study of Hodges *et al* on “A protocol for Safe Anaesthesia for cleft lip and palate surgery in developing countries” where similar technique was used only for patients from 1-10years old [10]. This study shows that the technique was tolerated well down to the age of 3 month. Ketamine and atropine, without intubation, were the only drugs used to anaesthetise 46(74.2%) patients who had cleft lip repair from the age of 3 month to 9 years and it proved to be efficient and safe. Regional anaesthesia was used for 14(22.6%) patients above the age of 9 years. Peri-incisional infiltration using cartridges of the dental preparation of lidocaine with adrenaline in 1.8ml was used. There was no form of sedation given to the patients unlike in the work of Hodges *et al* [10]. Most studies on regional anaesthesia for cleft lip repair used different combinations of infraorbital block, dorsal-nasal block, septal block, and peri-incisional infiltration [3, 8]. Only peri-incisional infiltration was used during this mission and patients tolerated it well and with good postoperative analgesia up to 6 hours. There were no complications recorded with this technique. This agrees with the findings of other studies [3, 8, 9, 10]. The acceptance level was high as there was no need to convert to general anaesthesia in any of the patients. General anaesthesia is the safest and preferred mode of anaesthesia for cleft surgeries because of the need to prevent aspiration while ensuring a good oxygenation of the patient [11, 12]. During this mission, general anaesthesia with endotracheal intubation could be performed in one patient at a time due to the availability of only one anaesthesia machine.

In addition, the continuous supply of oxygen could not be guaranteed. Hence, this technique was reserved for patients with bilateral cleft lip where more bleeding is expected, and to conserve oxygen for patients with complication that may require oxygen. There was adequate provision of the volatile anaesthetic agent, halothane, by the team but the agent could not be used due to the inadequate supply of oxygen and other equipment. There were no complications recorded in these patients. Studies on the use of general anaesthesia for cleft lip surgeries recorded some complications among which are hypoxia, laryngospasm, difficult intubation, failed intubation, temperature variation, tube disconnection, pulmonary oedema etc [12, 13]. The finding of no complication from this study could be due to the smaller number of patients who had this form of anaesthesia. Lower concentration of halothane (1% halothane in oxygen at 6L/min) was used while allowing the patients to breathe spontaneously because the anaesthesia was supplemented with peri-incisional infiltration of the dental preparation of lidocaine in adrenaline. Hence, the patients recorded faster recovery time. The mean duration of surgery was 34.2 minutes (median = 34.0mins). This duration is relatively shorter when compared to the work done by Jindal *et al* where the duration for cleft lip repair was one (1) hour and that of Eipe *et al* of between 45minutes to 60 minutes [8, 12]. This could be due to the fact that the team of surgeons and anaesthetist have been working together for more than 5 years on cleft surgeries and the experience gained over the years would have been contributory. Another factor could be the fact that every member of the team is a specialist including the anaesthetist with improvement in skill over time. Sierra Leone depends largely on the support of international organizations in their healthcare especially for surgical procedures [13]. Until 2008, there had not been any opportunity for postgraduate training in surgery in a country with only ten (10) trained surgeons to serve the whole population. The country also has shortages in infrastructure and supplies required for delivering surgical care [14]. Unlike other studies that recorded multiple complications with cleft lip surgeries, the only complication encountered during this mission is fever (Temperature > 37.5°C) which occurred in 6 (9.7%) patients [1, 5, 9, 12, 15]. Ironically, all 6 patients had ketamine anaesthesia. Similarly, the fever started in the intraoperative period and extended into the postoperative period. The fever was managed with acetaminophen syrup and patients were also treated for malaria since the country is a malaria endemic area. This study supports the findings of Fillies *et al* in their study on perioperative complications in infant cleft repair where temperature variation particularly hyperthermia accounted for most of the complications observed [13]. Quershi *et al* have reported hypothermia as an important complication found most commonly in children with a bilateral cleft lip repair citing prolonged duration of surgery as the cause [6]. However, in our study neither hypothermia nor hyperthermia was observed in the 2 (two) patients who had bilateral cleft lip repair under general anaesthesia.

## Conclusion

Most patients for cleft lip repair presents in the paediatric age group. This contributes to the challenge faced by anaesthetist apart from those presented by the pathology. Hence, general anaesthesia with endotracheal intubation is considered as the safest mode of anaesthesia. But our study has demonstrated that these surgeries can be performed with careful planning and with the required expertise in low resource setting.

### What is known about this topic

- That most African countries are lacking in adequate infrastructure needed for the repair of cleft lip and palate;
- That regional and general anaesthesia with endotracheal intubation are the two safe modes of anaesthesia for the repair of cleft lip.

### What this study adds

- That with extra caution and care, cleft lip can be safely repaired in a resource poor setting using ketamine anaesthesia;
- That peri-incisional infilteration alone, if properly done, can be used for cleft lip repair in adult.

## Competing interests

The author declare no competing interest.
